# A Method of Replacing Broken Facings on Crowns and Bridges without Removal from Mouth

**Published:** 1911-08-15

**Authors:** J. W. Bryan

**Affiliations:** Nashville, Tenn.


### A METHOD OF REPLACING BROKEN FACINGS ON CROWNS AND BRIDGES WITHOUT REMOVAL FROM MOUTH.

BY J. W. BRYAN, D.D.S., NASHVILLE, TENN.

Read before the Tennessee State Dental Association, May 23, 1911.

We are often called on to repair crown and bridge work because of a broken facing, or because the facing is an off color, or because of absorption of the gums.

To remove the crown or bridge is quite a task, and often results in practical destruction, and the repairing is often more troublesome than the making of a new piece. All these difficulties we want to obviate, and at the same time do a piece of wo k that will be efficient and esthetic, with the least possible inconvenience to patient and be the easiest on the operator.

In all such cases the platinum pins have been left attached to the metal backing. The first step is to cut off these platinum pins, and make the metal backing perfectly flat and smooth. Select a suitable facing of Steele's make and grind the gum end to fit as desired. Dry the metal backing of the bridge and put some warm wax on it, and then moisten the facing and press it into the desired position, and hold there until the wax has cooled. Then remove the facing by gently pressing it toward the incisal edge. It will slide off easily, leaving an elevation of the wax which fits into the tubular slot of the facing. Then using a small drill, made for this special use, pass through this wax ridge and mark the metal near each end of this ridge of wax where it is desired to drill holes in the metal backing. Remove the wax, and holding the drill perpendicular to the metal surface make a hole at the places marked, and as deep as desired. A drop of oil helps this part of the work very much.

Then these holes are tapped and a specially prepared gold screw is set into each hole. The heads of these screws passes easily into the tubular slot in the facing. The facing is now slipped on, to see that it fits properly. Remove the facing and screws and treat all parts with alcohol or chloroform, so as to have a clean dry surface for the cement.

The screws are then reset with cement and the facing again tried on to see that the screws are properly adjusted for the setting of the facing. Now remove the facing and treat the metal surface, the screw heads, and the facing with a smooth soft cement, and at once press the facing into its desired position, where it is to be held firmly until the cement sets.

This method of replacing the facing can usually be done in from fifteen to twenty-five minutes and gives results that are satisfactory and permanent, and is far more hygienic than the common methods afford, as no place is left between the backing and the porcelain for the lodgment of the secretions of the mouth, only as this cement washes out, and this occurs around inlays with equal or greater rapidity than here.

This, to me is by far the easiest and most satisfactory method of doing this work, and this bridge repair outfit is of such special service in other repair work that I would not like to be without it.

DISCUSSION.

Dr. Celia Rich: With many more of you I know the embarrassment of having facings broken from crowns and bridges, and if this method will simplify their repair or make their correction more readily accomplished, I shall welcome it. It seems to me in these two particulars, the rapidity with which the repair is made and the simplicity of the method make it very desirable. There are some broken facings, however, that I imagine could hardly be replaced in this way, those for instance having a protecting cap over the incisal edge.

Dr. A. C. Cottrell: I would like to ask Dr. Bryan if he has had any trouble in the breaking the drills and screws in trying to open up the holes for the reception of the screws? I have had much trouble with them myself, and instead of being twenty-five or thirty minutes I have spent as much as two or three hours. I have been using a method that is different from this, and I find in many cases that it is superior to this one. I select a suitable facing as you would in any ordinary case of bridge work, then bend the pins and bring them together and unite them with solder. If you want to you can make the pin longer or shorter. Then cut out a slot and pass the loup through it or into it far enough to get good anchorage. If the backing will admit, a groove can be cut in the lingual surface and a small bar passed through the loup and cemented to place will securely anchor the facing. The wire can be burnished down closely and concealed with the cement and the facing drawn up so securely to the backing that there will be no chance for moisture dissolving away the cement. I have repaired a great many facings in this way very satisfactorily when there was the necessary material and space to work in.

Dr. Henry W. Morgan: I have had some of Dr. Bryan's experience and also some of Dr. Cottrells'. When I first had my attention called to the method presented by Dr. Bryan, I thought this problem was solved, and I should never want anything better. You will find there are some people who are constantly breaking off the porcelain facings, no matter in what position you put them. There would be great advantage in replacing such broken facings if we could have a number of the facings on hand and when you have a necessity for one of them, you can make a proper selection and speedily make the needed repair.

As suggested by Dr. Cottrell, I have never been able to accomplish much in that way. I have had some cases that took two hours and a half and have had to take the reamer and cut out a place for the tooth.

Dr. E. Campbell: I would like to say that for those of us who live in towns where there are no dental supply houses we work very differently. When we use Steele's facings it is a very easy matter to select a tooth where we have a good many facings on hand, but I have never used it. When I have to put on a broken facing or crown or a porcelain tooth, I cut off the pins even with the porcelain and then with a diamond drill I drill down through into the porcelain and drill the pins out and cut the hole sufficiently large that the pins that remain in the backing may pass into the facing. Ordinarily I find that by grinding off these facing pins they will fit accurately and the heads of the pins are released.

Dr. Bryan: I am glad Dr. Cottrell spoke of his trouble

I haven't had the experience of breaking off the screw tops he mentions. Perhaps he has been trying to work too rapidly or in failing to use the kindly little drop of oil I suggested in aid of this work. In drilling the holes oft times the drill will make a quick cutting and because of this the drill clogs and becomes rigid, and in handling this all that can be done is to stop and blow out the cuttings. In so far as breaking the little tap referred to by Dr. Morgan when using the kind I have just referred to, I have not had that experience in this work. Sometimes the man who goes the slowest, gets along the fastest at his work. As Dr. Morgan has suggested, ofttimes if a patient is in a hurry you can get duplicate facings and in case the facing breaks, the patient can stop at another dentist's office and he will repair it. Every method appeals to me, especially since I have been satisfied that this has been so easily handled. Some of these cases are not very much trouble.

